# Human Hantavirus Infections in the Netherlands

**DOI:** 10.3201/eid2012.131886

**Published:** 2014-12

**Authors:** Jussi Sane, Johan Reimerink, Margriet Harms, Jacinta Bakker, Lapo Mughini-Gras, Barbara Schimmer, Wilfrid van Pelt

**Affiliations:** National Institute for Public Health and the Environment, Bilthoven, the Netherlands (J. Sane, J. Reimerink, M. Harms, J. Bakker, L. Mughini-Gras, B. Schimmer, W. van Pelt);; European Centre for Disease Prevention and Control, Stockholm, Sweden (J. Sane)

**Keywords:** hantavirus, viruses, Puumala virus, epidemiology, human infections, seroprevalence, voles, zoonoses, the Netherlands

## Abstract

We report the recent epidemiology and estimated seroprevalence of human hantavirus infections in the Netherlands. Sixty-two cases were reported during December 2008–December 2013. The estimated seroprevalence in the screened municipalities in 2006–2007 was 1.7% (95% CI 1.3%–2.3%). Findings suggest that hantavirus infections are underdiagnosed in the Netherlands.

Hantaviruses (family *Bunuyaviriade*, genus *Hantavirus*) are primarily rodent-borne pathogens that are a suspected cause of hemorrhagic fever with renal syndrome (HFRS) in Eurasia (*1*). They are transmitted to humans mainly through aerosolized rodent excreta (*1*). Five hantaviruses circulate among rodents in Europe, but most human HFRS cases are caused by Puumala virus (PUUV) (*1**,**2*). The reservoir for PUUV is bank voles (*Myodes glareolus*), which are widespread in Europe (*1**,**2*). HFRS is a reportable disease in most countries in Europe, and cases are reported mostly from Finland, Sweden, and forest-rich regions of Belgium and Germany (*1**,**3**,**4*).

In the Netherlands, hantavirus infections have been reportable since December 2008, although voluntary laboratory surveillance has been in place since 1989. An earlier study in the Netherlands reported a seroprevalence of 0.7% among blood donors but higher prevalences in forest workers and animal trappers (*5*). Antibodies to PUUV, Tula virus (TULV), and Seoul virus (SEOV) have been found in rodent populations in the Netherlands, and TULV has been isolated from common voles (*Microtus arvalis*) (*5**,**6*). The purpose of this study was to report recent trends in human hantavirus infection and estimate seroprevalence in the Netherlands.

## The Study

We analyzed reported data for the Netherlands for December 2008–December 2013. Reporting criteria included ≥1 hantavirus-associated symptom (fever, renal insufficiency, or thrombocytopenia) and virus detection in blood, or a major increase in IgG titers or increases in IgM or IgA titers against hantavirus. For the seroprevalence study, a subset of samples from a large serum bank established for population-based serologic studies (Pienter 2), particularly immunization program evaluations, was used. Pienter 2 is a cross-sectional serosurvey conducted during 2006–2007 with a representative sample (n = 7,904) of the population of the Netherlands (*7*).

**Figure 1:**
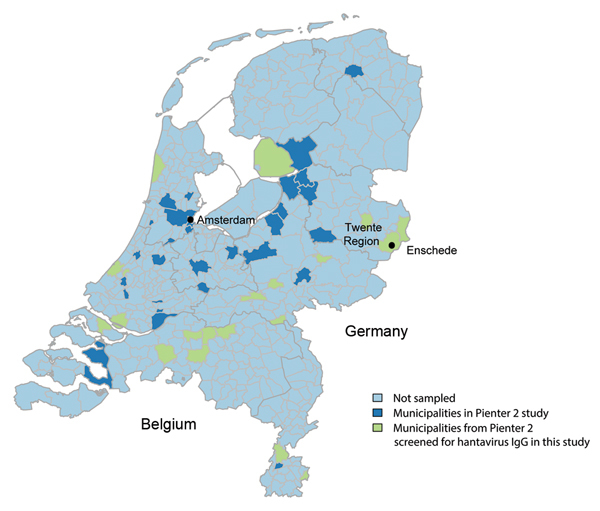
Municipalities sampled in the Pienter 2 study and subset of municipalities included in the seroprevalence study of hantavirus infections, the Netherlands.

Participants also completed a questionnaire that included basic demographic characteristics and behaviors and activities related to increased risk for acquiring infectious diseases. Variables possibly related to hantavirus infection from the literature, such as age, sex, outdoor activities, and animal contact, were selected from the Pienter 2 questionnaire. A total of 2,933 serum samples from 19 municipalities distributed across the country, including known high-risk areas, were included in the study and screened for antibodies against hantavirus (Figure 1).

An ELISA (Hantavirus IgG Dx Select; Focus Diagnostics, Cypress, CA, USA) that detects all known circulating hantaviruses in Europe was used for initial screening of all serum samples. For confirmation, all ELISA-positive samples were analyzed by using a PUUV-specific indirect immunofluorescence assay (IFA) (Progen, Heidelberg, Germany). Samples with reactivity at a dilution of 1:32 were considered positive for hantavirus infection. Manufacturer’s recommendations were followed for both assays. A randomly selected subset of ELISA-negative samples were screened by IFA to correct for possible false-negative ELISA results.

We calculated odds ratios (ORs) and 95% CIs for variables putatively associated with hantavirus seropositivity by using mixed-effects logistic regression that included municipality as a random effect to account for clustering of samples. All estimates were adjusted for age and sex. A p value ≤0.15 was used in the single-variable analysis to select variables for the multivariable model built in backward stepwise fashion. Statistical significance was considered at the 5% level.

**Figure 2:**
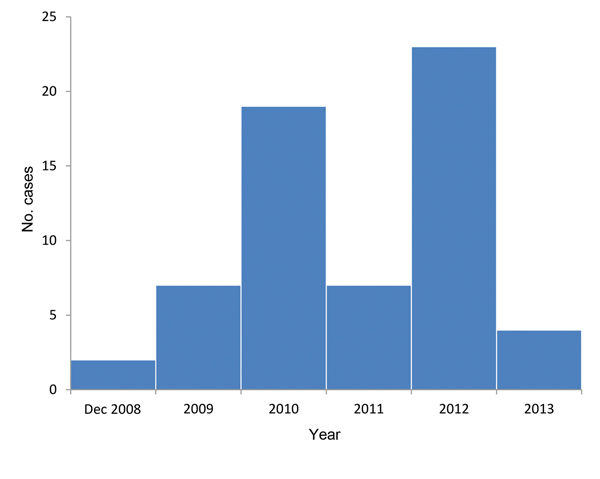
Reported cases of hantavirus infection (n = 62) by year, the Netherlands, December 2008–2013.

A total of 62 cases were reported during December 2008–December 2013 (Figure 2). Most cases (63.0%) were in men (median age 48 y, range 16–72 y). The highest number of cases (n = 26) occurred in the region of Twente (Figure 1) in the eastern Netherlands. Fifty-two case-patients (85.0%) were hospitalized and seven (12.0%) required dialysis. Most cases (90.0%) were acquired domestically.

A total of 154 (5.3%) serum samples were positive by ELISA. Of the ELISA-positive samples, 27 (17.5%) were also positive for PUUV IgG by IFA and therefore considered samples with positive results. One of the 119 ELISA-negative samples was also positive by IFA. After we corrected for false-negative results, the overall seroprevalence was 1.7% (95% CI 1.3%–2.3%).

Selected factors associated with PUUV infection are shown in the Table. Seroprevalence (uncorrected) in women was higher than that in men, albeit, not significantly. Age was not associated with PUUV infection. Owning ≥1 or more dogs or any livestock was associated with PUUV infection in a multivariable model (Table). Variables reflecting other outdoor activities were not associated with higher seroprevalence. The municipality-level random effect was significant (p = 0.001, by log-likelihood ratio test). Most positive samples (n = 10) were from the municipality of Enschede (Twente Region) in which seroprevalence was 3.2% (10/309) (Figure 1). Seroprevalence was lower (0.8%–1.8%) or 0% in all the other municipalities surveyed.

**Table:** Selected factors associated with positivity for IgG against Puumala virus, the Netherlands, December 2008–December 2013*

Factor	**No. persons IFA positive/no. tested (%)**	**Single variable**			**Multivariable**	
		aOR (95% CI)†	p value		aOR (95% CI)‡	p value
Sex						
M	9/1,368 (0.66)	Reference			Reference	NA
F	18/1,565 (1.15)	1.79 (0.89–3.57)	0.10		1.87 (0.97–3.61)	0.06
Age, y						
0–15	7/824 (0.84)	Reference			Reference	NA
16–40	7/822 (0.85)	1.03 (0.36–2.93)	0.96		1.0 (0.33–3.07)	0.99
41–60	5/585 (0.85)	0.91 (0.26–3.19)	0.88		0.86 (0.22–3.32)	0.82
>60	8/702 (1.14)	1.32 (0.48–3.62)	0.59		1.69 (0.51–5.56)	0.39
Owning ≥1 dog						
No	12/2147 (0.56)	Reference			Reference	NA
Yes	15/786 (1.53)	4.51 (1.81–11.30)	0.001		3.49 (1.50–8.14)	0.004
Owning any livestock§						
No	19/2,706 (0.70)	Reference			Reference	NA
Yes	8/227 (3.52)	6.97 (2.45–19.82)	<0.001		4.79 (1.69–13.57)	0.003
Net monthly income, Euros						
<1,150	11/426 (2.58)	Reference			NA	NA
1,151–3,050	9/1,539 (5.84)	0.27 (0.05–1.47)	0.13		NA	NA
>3,501	1/242 (0.41)	0.21 (0.03–1.79)	0.15		NA	NA
Occupational exposure to any animal						
No	25/2794 (0.89)	Reference			NA	NA
Yes	2/139 (1.43)	1.72 (0.25–11.77)	0.58		NA	NA

*IFA, indirect immunofluorescence assay; aOR, adjusted odds ratio; NA, not applicable. The municipality-level random effect was significant (p = 0.001, by log-likelihood ratio test). †Adjusted for age, sex, and clustering at municipality level (random effect).  ‡Adjusted for age, sex, clustering at municipality level (random effect), and the other covariates included in the multivariable model. §Cattle, pigs, sheep, goats, poultry, and other livestock.

## Conclusions

We report that hantavirus seroprevalence in the Netherlands is 1.7% for the years analyzed on the basis of a subset of samples from a large population-based serum bank. This seroprevalence was similar to estimates for neighboring countries (1.5% for Belgium and 1%–3% for Germany) (*2**,**4*). However, comparing prevalences is challenging because of different methods used and populations studied.

The number of reported cases in the Netherlands was low. Given our seroprevalence estimate, although not entirely representative of the population of the Netherlands, and the proportion of symptomatic PUUV-infected persons (20%–30%), some of whom seek medical care (*1*), the number of cases in the Netherlands (≈16.5 million resident population) is expected to be higher. Many cases with milder symptoms probably go unnoticed because of low awareness of hantavirus infection among physicians in the Netherlands. Hantavirus infections in the Netherlands are most likely caused by PUUV, although SEOV- or TULV-associated infections cannot be excluded; clinical SEOV infections have been reported from France and the United Kingdom (*8**,**9*).

The proportions of hospitalizations and persons requiring dialysis were much higher than those reported in other countries. In Finland, 52.0% of PUUV-infected persons required hospitalization (*10*). In Germany, 64.0% of persons with reported cases of infection in 2010 required hospitalization (*11*). The difference in hospitalization rates is probably associated with reporting bias because only the most severe cases are reported due to strict reporting criteria and possible higher thresholds for testing. This finding also suggests that a large number of milder cases are being underreported.

Bank voles are a forest-dwelling species, and risk for PUUV infection is associated with vicinity of forests and the proportion of forested land cover (*1**,**12**,**13*). Seroprevalence and number of cases were highest in the Twente Region, a region to which PUUV is endemic. This region borders areas in Germany in which incidence is high (*11*) and is located near forests, which are scarce in the Netherlands. The only variables associated with PUUV infection were dog and livestock ownership. However, it is highly unlikely that these factors reflect direct virus transmission from such domestic animals to humans, but represent proxies for lifestyle characteristics of dog owners and persons engaged in farming activities that predispose them to more frequent or more substantial contact with rodents.

Seroprevalence was higher in women, but the disease was most often reported in men. Similar male:female ratios among persons with clinical PUUV infection have been reported from other countries (*10**,**14*). These data suggest that women have a higher proportion of subclinical or mild infections, although a recent study suggested that disease severity does not differ between men and women (*15*).

In conclusion, higher seroprevalence relative to the number of reported cases calls for further awareness of hantavirus infection among physicians in the Netherlands. Seroprevalence studies of persons with unresolved renal problems could further increase our understanding of the true incidence of hantavirus infection in the Netherlands.
